# Meta-Analysis of Mitochondrial DNA Variation in the Iberian Peninsula

**DOI:** 10.1371/journal.pone.0159735

**Published:** 2016-07-21

**Authors:** Ruth Barral-Arca, Sara Pischedda, Alberto Gómez-Carballa, Ana Pastoriza, Ana Mosquera-Miguel, Manuel López-Soto, Federico Martinón-Torres, Vanesa Álvarez-Iglesias, Antonio Salas

**Affiliations:** 1 Unidade de Xenética, Departamento de Anatomía Patolóxica e Ciencias Forenses, Instituto de Ciencias Forenses, Facultade de Medicina, Universidade de Santiago de Compostela, Galicia, Spain; 2 GenPop Research Group, Instituto de Investigaciones Sanitarias (IDIS), Hospital Clínico Universitario de Santiago, Galicia, Spain; 3 Grupo de Investigación en Genética, Vacunas, Infecciones y Pediatría (GENVIP), Hospital Clínico Universitario and Universidade de Santiago de Compostela (USC), Galicia, Spain; 4 Servicio de Biología, Instituto Nacional de Toxicología y Ciencias Forenses, Departamento de Sevilla, Sevilla, Spain; 5 Pediatric Emergency and Critical Care Division, Department of Pediatrics, Hospital Clínico Universitario de Santiago, Santiago de Compostela, Galicia, Spain; Universita degli Studi di Pavia, ITALY

## Abstract

The Iberian Peninsula has been the focus of attention of numerous studies dealing with mitochondrial DNA (mtDNA) variation, most of them targeting the control region segment. In the present study we sequenced the control region of 3,024 Spanish individuals from areas where available data were still limited. We also compiled mtDNA haplotypes from the literature involving 4,588 sequences and 28 population groups or small regions. We meta-analyzed all these data in order to shed further light on patterns of geographic variation, taking advantage of the large sample size and geographic coverage, in contrast with the atomized sampling strategy of previous work. The results indicate that the main mtDNA haplogroups show primarily clinal geographic patterns across the Iberian geography, roughly along a North-South axis. Haplogroup HV0 (where haplogroup U is nested) is more prevalent in the Franco Cantabrian region, in good agreement with previous findings that identified this area as a climate refuge during the Last Glacial Maximum (LGM), prior to a subsequent demographic re-expansion towards Central Europe and the Mediterranean. Typical sub-Saharan and North African lineages are slightly more prevalent in South Iberia, although at low frequencies; this pattern has been shaped mainly by the transatlantic slave trade and the Arab invasion of the Iberian Peninsula. The results also indicate that summary statistics that aim to measure molecular variation, or AMOVA, have limited sensitivity to detect population substructure, in contrast to patterns revealed by phylogeographic analysis. Overall, the results suggest that mtDNA variation in Iberia is substantially stratified. These patterns might be relevant in biomedical studies given that stratification is a common cause of false positives in case-control mtDNA association studies, and should be also considered when weighting the DNA evidence in forensic casework, which is strongly dependent on haplotype frequencies.

## Introduction

Analysis of mitochondrial DNA (mtDNA) variation has become a convenient tool for the analysis of population substructure. Population structure is commonly identified when samples have deviations from Hardy-Weinberg proportions, or deviations from panmixia. Conditions such as behavioral restrictions of mating, inbreeding, selection, or migration are the main causes of population substructure, since they are responsible for potential mates not having an equal chance of being selected as partners. This definition, however, does not apply to mtDNA variation because this marker is inherited in a matrilineal way. Since mtDNA does not suffer from recombination, there is no possibility to calculate the frequency of composite genotypes from their component frequencies, as typically done with autosomal markers [1]. The detection of differences in haplotype frequencies has the main limitation that large sample sizes are usually needed. Phylogeographic analysis can help reveal the existence of particular haplogroups or haplotypes that are more prevalent in specific regions.

Many previous studies were not particularly conceived for the detection of population substructure. Moreover, differences between neighboring populations are generally gradual and smooth, and samples may not be representative of the entire variation existing in a given population. Even in apparently continuous populations, different areas may have different gene frequencies, because the entire meta-population may not necessarily be panmictic (there is no random mating, there are mating restrictions, either genetic or behavioral). Some minor ethnic group may also show differences with the main population group; this is, for example, the case of the Romani population in the Iberian Peninsula [2].

Population structure is relevant not only in evolutionary genetics, but also in forensic genetics and biomedical studies [1,3]. For example, forensic laboratories typically use small, local databases in casework. Since mtDNA displays very high variability and is very sensitive to genetic drift, a database of e.g. 100 individuals may represent a small fraction of the variation only; as a consequence, estimates of haplotype frequencies might be subjected to great uncertainty [1,4,5]. On the other hand, correction of population stratification in forensic casework using statistical indices (e.g. *F*_*ST*_) might be inappropriate depending on the population context [6]. Mitochondrial DNA stratification is also an essential issue in biomedical studies, and it is in fact the cause of a large number of false positives in case-control association studies [7–9].

The Iberian Peninsula has been the focus of interest of a number of population mtDNA studies since more than 20 years ago. The Basque Country (North of Spain) was the focus of some of the pioneering studies [10]. This region has attracted the attention of numerous scientists due to the fact that the Cantabrian refuge is supposed to retain genetic signatures of the human population retractions occurred during the Last Glacial Maximum (LGM) [11–14]. Corte-Real et al. [15] sequenced the mtDNA hypervariable region I (HVS-I) of individuals recruited in different locations of Iberia, including the Basque Country. Their results indicated some distinctive features in the Basque compared to other Iberian regions. Galicia (Northwest of the Iberian Peninsula) has also been the focus of interest because historically this region was relatively isolated from the rest of the Iberian Peninsula, with almost no recent immigration [16,17]. Like the Basque Country, Galicia preserved a strong cultural identity and a distinct language; its genetics patterns show also signatures of a cul-de-sac population expansion. Over the last few years, several other studies have analyzed many other regions of Spain, including Zamora [18], Navarre [19], Catalonia [20], the Atlantic Facade [21], the Mediterranean [22], etc.

The genetic characterization of other geographically isolated populations in Iberia has received the interest of various studies. Thus, for instance, Larruga et al. [23] analyzed the Maragato population; their study allowed them to refute their historically presumed Berber origin. The isolated population of Pasiegos (Cantabrian region) was the focus of interest in the study by Maca-Meyer et al. [24]; this population also showed signatures of strong isolation, probably as a result of the rough orography coupled with other historical circumstances (e.g. their historically documented resistance to numerous waves of invaders).

The Romani community of Iberia has also been analyzed in the literature by Gresham et al. [25], and more recently by Moorjani et al. [26] and Gómez-Carballa et al. [2]. Besides exploring their patterns of genomic admixture using different sets of markers, these studies were able to trace clear signatures of their Indian ancestry.

Portugal has received the attention of several mtDNA studies. Pereira et al. [27] reported a higher level of diversity in comparison with the surrounding populations. These authors noted the presence of all the most important European haplogroups, but also the presence of African haplogroups probably originating at the time of the transatlantic slave trade.

Spanish and Portuguese islands have also been sampled and analyzed for mtDNA, including the Canary Islands [28], Majorca in the Balearic Islands [29,30], and Madeira and Açores [28,31,32].

Given the very large number of studies focused on Iberia during the last two decades, there is now an opportunity to meta-analyze these data to investigate patterns of geographic structure. Thus, the present study aims to analyze patterns of mtDNA diversity in the Iberian Peninsula by way of compiling all available control region data in the literature since about 1995 (4588). In addition, we provide newly generated data (3024 control region sequences) covering additional regions of the Iberian Peninsula, including some important regions (e.g. Andalusia) were sample sizes were still comparatively small. We sought patterns of population stratification that might have implications in various fields of biomedical and forensic research. Furthermore, in our large DNA data bank, we selected eight individuals that displayed uncommon haplotypes in their control region for complete mtDNA sequencing, with the aim of characterizing new branches of the global mtDNA phylogeny.

## Methods

### Samples

For the present study we analyzed 3,024 DNA samples from the Spanish National DNA Bank project (http://www.bancoadn.org). This DNA Bank is a service that receives, processes and stores DNA from voluntary Spanish donors along with relevant information on health and lifestyle habits related to the samples. These samples are made available to the scientific community with the aims of facilitating, promoting and developing national and international scientific research on human evolution, genetic/genomic diversity with regard to health, and origins and treatment of illnesses. For all the donors, the National DNA Bank records information on the geographic origin of the maternal ancestors (including mother and grandmother).

### Ethics Statement

Written informed consent is obtained by the National DNA Banks for all the donors. See http://www.bancoadn.org for more information on ethic procedures on sample collection of the Spanish National DNA Bank.

### PCR and mtDNA sequencing

PCR amplification was performed in a 9700 Thermocycler (AB) using 32 cycles of amplification and the following temperature profile: 95°C for 30 s, 58°C for 1m30 s, and 72°C for 1m30 s The control region was sequenced in a total of 3,024 samples for a variable range that for most of the samples covers from position 16024 to about the 130 first bases of the HVS-II (S1 Table). Sequencing protocol of the control region and the mitogenomes was as previously described [12,33,34]. Mitogenomes have GenBank accession numbers KX055565–KX055572.

### Statistical analysis

We compiled a total of 4,588 sequences from the literature (S2 Table). Most of them contain information on the HVS-I only, while 2037 contain additional data on the HVS-II. We merged these data with the new data generated for the present study, adding up to a grand total of 7,611 control region sequences. Exploratory inference of haplogroup status of these sequences was carried out using Haplogrep [35]. This initial haplogroup classification was however contrasted with the latest version of Phylotree and manually checked [36].

For most computations, only the common segment that ranges from position 16024 to position 16362 was used. Depending on the purpose of the analysis, haplogroup status was inferred (*i*) using all the sequence information available, and (*ii*) using the common sequence segment available for all the haplotypes in the database. Although the latter procedure leads to a loss of phylogenetic resolution, it allows the inference of spatial patterns of haplogroup geographic variation using a homogeneous haplogroup classification for all available data.

**Fig 1:**
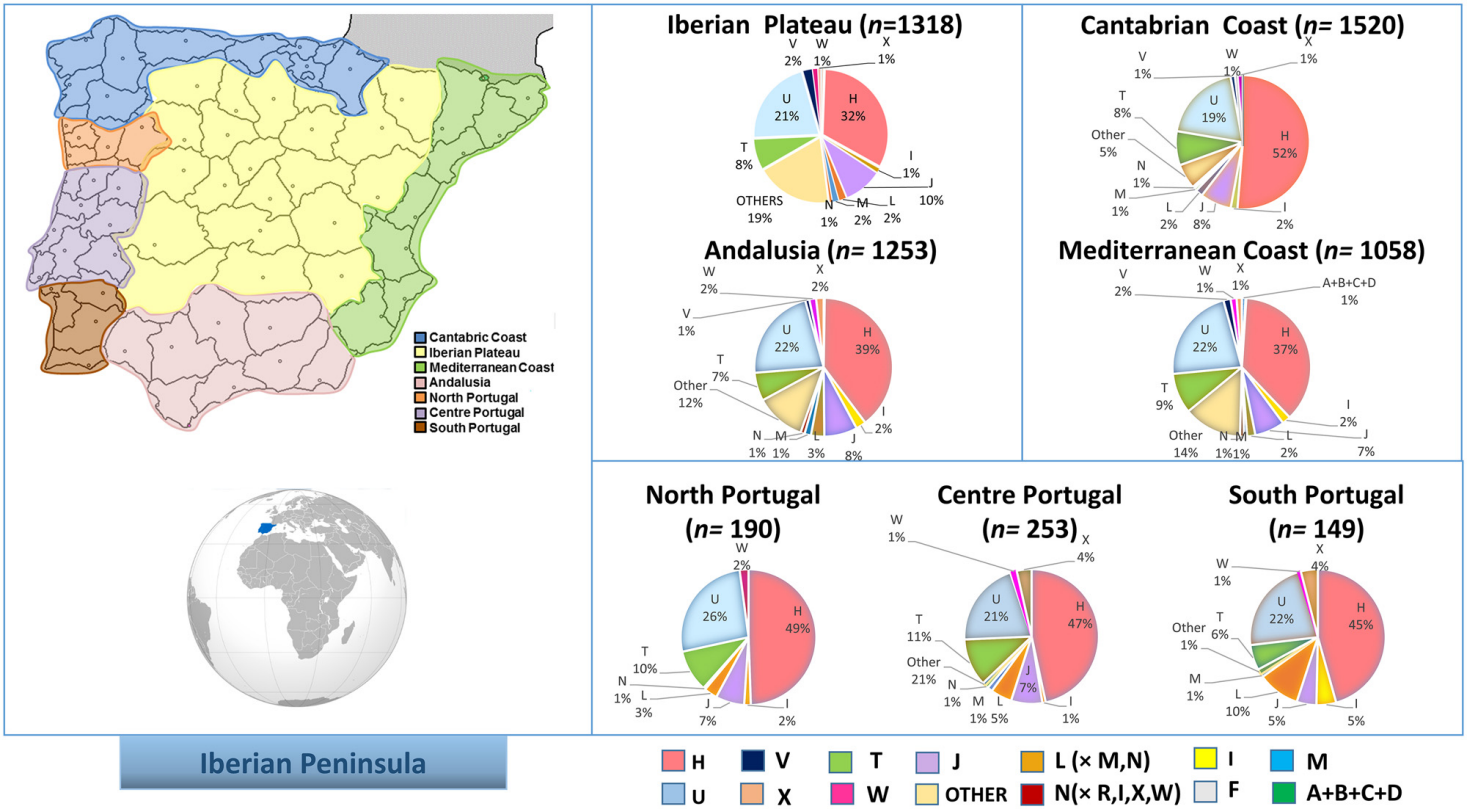
Map showing the location of the main Iberian regions analyzed in the present study. The pie charts show the frequency values for the main haplogroup categories in these main Iberian regions. Note that the category “Other” in the pie charts denotes an artificial polyphyletic cluster (paragroup). Other haplogroup labels represent the following haplogroup categories: L = L(× M,N); N = N(×R,I,X,W).

In order to explore possible patterns of variation across the Iberian Peninsula, various grouping schemes based on the geographic units and political borders were evaluated, and several computations were carried out according to these different clustering criteria. Thus, we divided the Iberian Peninsula in seven main regions, namely, Cantabrian Coast, Mediterranean, Iberian Plateau, Andalusia, and North, Central and South Portugal (Fig 1). Subdivision of Portugal in three main regions is justified on historical and geographic criterion. Thus, North, Central and South Portugal were divided by the natural boundaries that represent the two main rivers crossing the country, namely, Douro and Tagus. Traditionally the North of Portugal has had a strong relationship with the northwest Spanish region of Galicia, since during the whole medieval era they shared a common language (Galician-Portuguese) and a common cultural background (a relationship that still exists today). Remarkably, the South of Portugal has been signaled as one of the main regions that contributed more actively to the Transatlantic Slave Trade. Most of previous genetic studies used this sub-division (see e.g. [21,37,38]).

Spanish samples were also grouped in 17 politically defined “Autonomous Regions”. Finally, analyses were additionally carried out by politically defined provinces.

Diversity indices computed on mtDNA sequences were obtained using DnaSP v.5. [39]. Genetic structure and variation between individual and grouped population sets were carried out by means of Analysis of Molecular Variance (AMOVA) and RST genetic distances as implemented in Arlequin software v3.5.1.2 [40].

Spatial geographic representation of haplogroup frequencies was carried out using SAGA v. 2.1.1 (http://www.saga-gis.org/). We followed the commonly used ordinary Kriging method for interpolating frequency values (other interpolated methods yielded virtually the same results).

Mitogenomes belonging to haplogroups H14a and H14a2 were compiled from the literature and public databases (S3 Table); mtDNA data obtained from ancient specimens were not used. Phylogenetic reconstruction of haplogroups H14a and H14a2 was carried out by building maximum parsimony trees. Hotspot mutations such as 16182C, 16183C, and 16519C were not considered.

The Time of the Most Recent Common Ancestor (TMRCA) of haplogroup H14a was computed using a maximum likelihood procedure and following the phylogeny of Phylotree. For this purpose, the software PAML 4.4.[41] was used assuming the HKY85 mutation model (ignoring indels, as per common practice) and using gamma-distributed rates (approximated by a discrete distribution with 32 categories) and three partitions: HVS-I (positions 16051–16400), HVS-II (positions 68–263), and the coding region. Hotspot mutations such as 16182C, 16183C and 16519 were excluded from the computation. Age estimates were extrapolated using the corrected mutation rate of Soares et al. [42].

## Results

### Molecular diversity

Table 1 summarizes the main molecular diversity values for the regions covered in the present study, as well as sample sizes. Within the Iberian Peninsula, Spain has overall higher values of haplotype diversity than Portugal. Andalusia and the Iberian Plateau, followed by the Mediterranean region, have the highest values within the Peninsula. Nucleotide diversity has its highest value in the Mediterranean area, but it is also high in Portugal. When examined by Spanish Autonomous Regions, it is La Rioja, in the North of the Iberian Plateau, that has the highest values of haplotype diversity, followed by Extremadura in the South. Nucleotide diversity shows the highest levels in Navarra (North), and is clearly lower in the neighboring Basque Country (North). In the Balearic and Canary Islands, both haplotype and nucleotide diversity values are comparable with those in mainland Spain and Portugal (Table 2).

**Table 1:** Summary statistics of HVS-I sequences in Spain and Portugal (excluding islands) carried out on main geographic regions. All the computations were carried out on the common sequence segment of the HVS-I that ranges from 16024 to 16362. Population codes are as follows: a) Cantabrian coast = Galicia + Asturias + Cantabria + Basque Country + Navarra; b) Iberian Plateau = Castilla y León + Castilla La Mancha + La Rioja + Extremadura + Madrid + Aragón; c) Mediterranean coast = Murcia + Valencia + Catalonia; d) Andalusia; e) North Portugal = Viana do Castelo + Braga + Vila Real + Porto + Braganca; f) Centre Portugal = Aveiro + Viseu + Guarda + Coimbra + Castelo Branco + Leiria + Lisboa + Santarem + Portalegre; and g) South Portugal = Setubal + Evora + Beja + Faro. Note that sum of sample sizes of population sub-sets do not fully match with the total numbers mentioned above (4,588 from the literature + 3024 newly generated in the present study) because there was not enough geographic information for a few of them (the same applies to Tables 2 and 3).

Population	*n*	k	k/*n*	S	*N*mut	H ± SE	Π ± SE	M
**Cantabrian coast**	1520	449	0.30	152	180	0.9639±0.0030	0.01132±0.00103	3.83
**Iberian Plateau**	1336	489	0.37	144	164	0.9723±0.0029	0.01218±0.00200	4.10
**Mediterranean coast**	1063	430	0.41	175	220	0.9677±0.0050	0.01421±0.00093	4.84
**Andalusia**	1253	463	0.37	175	213	0.9720±0.0030	0.01286±0.00037	4.36
**North Portugal**	190	110	0.58	81	86	0.9460±0.0130	0.01288±0.00070	4.37
**Centre Portugal**	253	142	0.56	96	103	0.9630±0.0080	0.01324±0.00060	4.49
**South Portugal**	149	91	0.61	72	74	0.9560±0.0130	0.01363±0.00098	4.62
**Spain**	6021	1344	0.22	232	351	0.9675±0.0010	0.01216±0.00080	4.07
**Portugal**	1591	478	0.30	168	207	0.9650±0.0030	0.01402±0.00095	4.74

*n* = Sample Size; *k* = Number of different haplotypes; *S* = number of polymorphic (segregating sites); Nmut = total number of mutations; *H* = haplotype diversity and standard error; *π* = nucleotide diversity and standard error; *M* = Average number of nucleotide differences

**Table 2:** Summary statistics of HVS-I sequences in Spain and Portugal (excluding islands) carried out on main political regions (“Autonomous Regions”). All the computations were carried out on the common sequence segment of the HVS-I that ranges from 16024 to 16362.

Population	*n*	k	k/*n*	S	*N*mut	H ± SE	Π ± SE	M
**Andalusia**	1253	463	0.37	175	213	0.972±0.003	0.01286±0.00037	4.36
**Aragón**	78	55	0.71	56	56	0.969±0.013	0.01132±0.00092	3.84
**Asturias**	112	72	0.64	63	65	0.978±0.008	0.00800±0.00074	3.81
**Islas Baleares**	273	128	0.47	89	91	0.974±0.006	0.01314±0.00049	4.45
**Islas Canarias**	343	153	0.44	107	114	0.965±0.006	0.01481±0.00052	5.02
**Cantabria**	438	133	0.30	122	139	0.968±0.004	0.01201±0.00082	4.07
**Castilla y León**	671	304	0.45	124	139	0.971±0.004	0.01222±0.00034	4.15
**Castilla La Mancha**	256	146	0.57	103	106	0.976±0.006	0.01295±0.00059	4.39
**Catalonia**	731	417	0.44	195	205	0.985±0.002	0.01480±0.00055	5.27
**Valencia**	200	121	0.61	84	88	0.970±0.008	0.01215±0.00060	4.12
**Extremadura**	92	69	0.76	60	64	0.980±0.009	0.01276±0.00080	4.33
**Galicia**	548	256	0.47	161	197	0.961±0.006	0.01267±0.00090	4.30
**Madrid**	218	121	0.56	87	80	0.972±0.006	0.01190±0.00060	4.03
**Murcia**	134	83	0.62	62	63	0.962±0.012	0.01227±0.00075	4.16
**Navarra**	137	56	0.41	126	131	0.957±0.009	0.01576±0.00341	5.34
**Basque Country**	285	111	0.39	77	77	0.942±0.010	0.00940±0.00043	3.19
**La Rioja**	20	17	0.85	21	21	0.982±0.026	0.01045±0.00118	3.54

*n* = Sample Size; *k* = Number of different haplotypes; *S* = number of polymorphic (segregating sites); Nmut = total number of mutations; *H* = haplotype diversity and standard error; *π* = nucleotide diversity and standard error; *M* = Average number of nucleotide differences

Diversity was also computed to a smaller geographical scale, namely, by Spanish provinces (Table 3). Some of these values are not comparable due to their small sample size; this problem affects mainly the North African Spanish provinces of Ceuta and Melilla, and Bizkaia (in the Basque Country). Excluding these provinces, the highest values of haplotype diversity were found in the northern provinces of the Iberian plateau, namely, Zamora and Valladolid, while the lowest values were observed also within the Iberian Plateau. The highest nucleotide diversity was also observed in Zamora, whereas the lowest value was found in Barcelona.

**Table 3:** Summary statistics of HVS-I sequences in Spain (including islands) carried out on Spanish provinces. All the computations were carried out on the common sequence segment that ranges from 16024 to 16362.

Population	*n*	K	k/*n*	S	*N*mut	H ± SE	Π ± SE	M
**Ceuta**	7	5	0.71	10	10	0.905±0.103	0.01011±0.00338	3.43
**Melilla**	7	7	1.00	11	11	1.000±0.076	0.01096±0.00173	3.71
**Albacete**	45	35	0.78	44	44	0.982±0.982	0.01241±0.00120	4.21
**Alicante**	51	36	0.71	41	42	0.971±0.013	0.01110±0.00113	3.76
**Almería**	66	43	0.65	48	52	0.979±0.009	0.01427±0.00119	4.84
**Asturias**	112	72	0.64	63	65	0.978±0.008	0.00800±0.00074	3.81
**Ávila**	35	26	0.74	40	42	0.950±0.030	0.01075±0.00140	6.24
**Badajoz**	46	41	0.89	40	42	0.989±0.989	0.01246±0.00095	4.22
**Islas Baleares**	273	128	0.47	89	91	0.974±0.006	0.01314±0.00049	4.45
**Barcelona**	280	164	0.59	123	140	0.973±0.006	0.00600±0.00177	5.33
**Bizkaia**	9	9	1.00	17	17	1.000±0.052	0.01295±0.00249	4.39
**Burgos**	37	28	0.76	40	40	0.964±0.022	0.01154±0.00159	3.91
**Cáceres**	44	37	0.84	46	49	0.982±0.982	0.01363±0.01363	4.62
**Cádiz**	101	66	0.65	58	60	0.977±0.008	0.01285±0.00087	4.36
**Cantabria**	438	133	0.30	122	139	0.968±0.004	0.01201±0.00082	4.07
**Castellón**	22	17	0.77	25	25	0.974±0.022	0.01208±0.00173	4.10
**Ciudad Real**	49	39	0.80	49	50	0.984±0.010	0.01413±0.00173	4.79
**Córdoba**	214	117	0.55	128	142	0.972±0.006	0.01324±0.00186	4.49
**A Coruña**	40	33	0.83	43	43	0.964±0.023	0.01129±0.00124	3.83
**Cuenca**	49	40	0.82	42	42	0.980±0.013	0.01243±0.00108	4.21
**Gipúzkoa**	11	9	0.82	16	16	0.964±0.051	0.01148±0.00199	3.89
**Gerona**	39	25	0.64	33	33	0.950±0.024	0.01010±0.00111	3.42
**Granada**	133	81	0.61	67	69	0.978±0.007	0.01157±0.00068	3.92
**Guadalajara**	27	25	0.93	32	32	0.991±0.014	0.01434±0.00151	4.86
**Huelva**	66	40	0.61	47	48	0.961±0.016	0.01329±0.00117	4.50
**Huesca**	27	21	0.78	35	35	0.966±0.025	0.01163±0.00157	3.94
**Jaén**	154	95	0.61	80	86	0.957±0.012	0.01242±0.00073	4.21
**León**	162	86	0.53	70	74	0.939±0.016	0.01071±0.00069	3.63
**Lleida**	72	46	0.64	51	52	0.948±0.020	0.01227±0.00112	4.16
**Lugo**	22	18	0.82	28	28	0.983±0.018	0.01037±0.00135	3.52
**Madrid**	218	121	0.56	87	80	0.972±0.006	0.01190±0.00060	4.03
**Málaga**	163	99	0.60	81	85	0.972±0.008	0.01245±0.00068	4.22
**Murcia**	134	83	0.62	62	63	0.962±0.012	0.01227±0.00075	4.16
**Navarra**	137	56	0.41	126	131	0.957±0.009	0.01576±0.00341	5.34
**Ourense**	18	16	0.89	21	21	0.987±0.023	0.01124±0.00139	3.81
**Palencia**	24	21	0.88	32	33	0.986±0.018	0.01219±0.00162	4.13
**Las Palmas**	13	12	0.92	28	28	0.987±0.035	0.01528±0.00283	5.18
**Pontevedra**	46	37	0.80	45	45	0.977±0.015	0.01345±0.00117	4.56
**La Rioja**	20	17	0.85	21	21	0.982±0.026	0.01045±0.00118	3.54
**Salamanca**	182	103	0.56	74	79	0.976±0.006	0.01208±0.00059	4.10
**Santa Cruz de Tenerife**	2	2	1.00	4	4	1.000±0.500	0.01180±0.00590	4.00
**Segovia**	42	27	0.64	32	32	0.941±0.028	0.01059±0.00106	3.59
**Sevilla**	133	93	0.70	73	74	0.984±0.006	0.01336±0.00073	4.53
**Soria**	17	12	0.71	13	13	0.949±0.037	0.00829±0.00147	2.81
**Tarragona**	58	41	0.71	51	51	0.972±0.014	0.01171±0.00106	3.97
**Teruel**	20	16	0.80	22	22	0.974±0.025	0.01082±0.00140	3.67
**Toledo**	49	37	0.76	49	50	0.965±0.019	0.01276±0.00138	4.32
**Valencia**	125	86	0.69	106	116	0.970±0.011	0.01490±0.00255	5.05
**Valladolid**	30	27	0.90	36	38	0.993±0.011	0.01385±0.00149	4.69
**Zamora**	142	113	0.80	81	84	0.992±0.003	0.01520±0.00078	5.15
**Zaragoza**	30	25	0.83	32	32	0.975±0.021	0.01134±0.00148	3.84

*n* = Sample Size; *k* = Number of different haplotypes; *S* = number of polymorphic (segregating sites); Nmut = total number of mutations; *H* = haplotype diversity and standard error; *π* = nucleotide diversity and standard error; *M* = Average number of nucleotide differences

Diversity was also computed for the Atlantic Portuguese islands. Also here we found very high haplotype and nucleotide diversity values.

**Fig 2:**
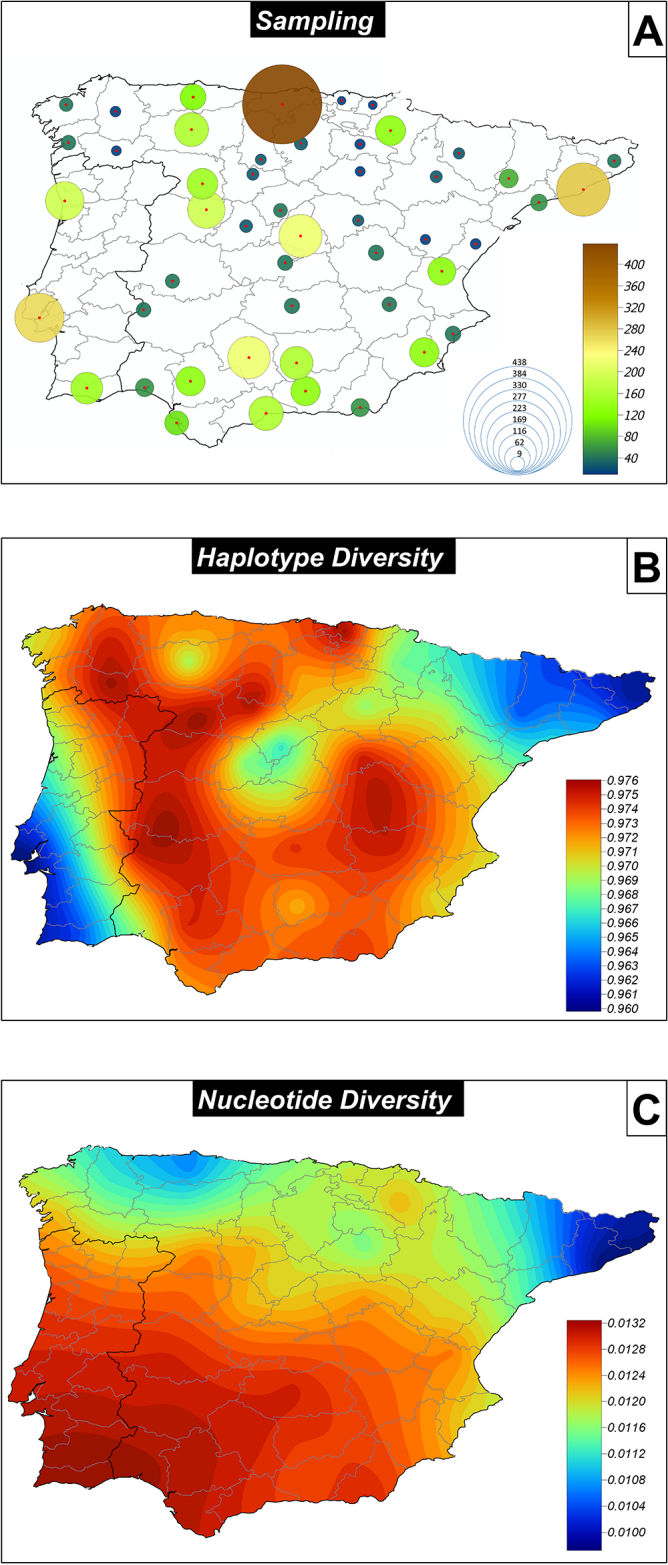
Geographic maps of sample sizes (A) and diversity indices (B and C).

A visual impression of Iberian mtDNA diversity patterns can be obtained by way of building maps of interpolated haplotype and nucleotide diversities (Fig 2). These maps show that haplotype diversity is lower in the southwest and the northeast corners of the Iberian Peninsula, but quite homogeneous in the rest of the Iberian territory. Nucleotide diversity shows a more clinal pattern that runs from high values in the South to decreasing values northwards.

### Patterns of spatial variation of haplogroup frequencies

In order to allow comparisons between haplogroup frequencies in different Iberian regions, a common sequence segment was used for haplogroup classification of the full database. Haplogroups were additionally collapsed into main haplogroup categories that represent the main European phylogenetic branches. The most frequent category is haplogroup H (Fig 1). The frequency estimated for haplogroup H might represent an underestimation, given that the control region alone provides limited phylogenetic resolution. The patterns observed are however very informative of the population stratification existing in the Iberian Peninsula. The estimated frequency of haplogroup H ranges from 52% in the Cantabrian Coast to 32% in the Iberian Plateau (and 37%-39% in the Mediterranean and Andalusia); Fig 1. The high frequency of haplogroup H in the North of Iberia spans all along the Portuguese territories, where frequencies range from 49% in the North to 45% in the South. Haplogroup U constitutes the second most frequent haplogroup in Iberia; its frequency is similar in the main Iberian regions; it reaches the highest frequency in North of Portugal (26%). Differences for the rest of the haplogroup categories are not so marked when examined by main geographic regions. Some differential pattern is more apparent when examining the data by political regions (S1 Fig). Again, the regions in the North (Basque Country, Cantabria, and Galicia) show the highest haplogroup H frequencies. Haplogroup U reaches 27% in the community of Madrid and 17% in the Basque Country, Valencia and Aragón; the lowest value was found in La Rioja (5%; although here the sample size is small). The polyphyletic cluster A+B+C+D represents the Native American ancestry existing in Iberia, mainly explained by modern South and Central American (Ecuador Colombia, Bolivia, Peru, etc.) immigration into Iberian territories (Instituto Nacional de Estadística: http://www.ine.es). The Native American ancestry of this collapsed category is coherent with the fact that it is only present in Iberia and virtually absent in Portugal (where immigration of Native American communities is much lower than in Spain; Instituto Nacional de Estatística: https://www.ine.pt/). Finally, sub-Saharan L-haplogroups are slightly more frequent in Andalusia (3%) than in the other Iberian regions.

**Fig 3:**
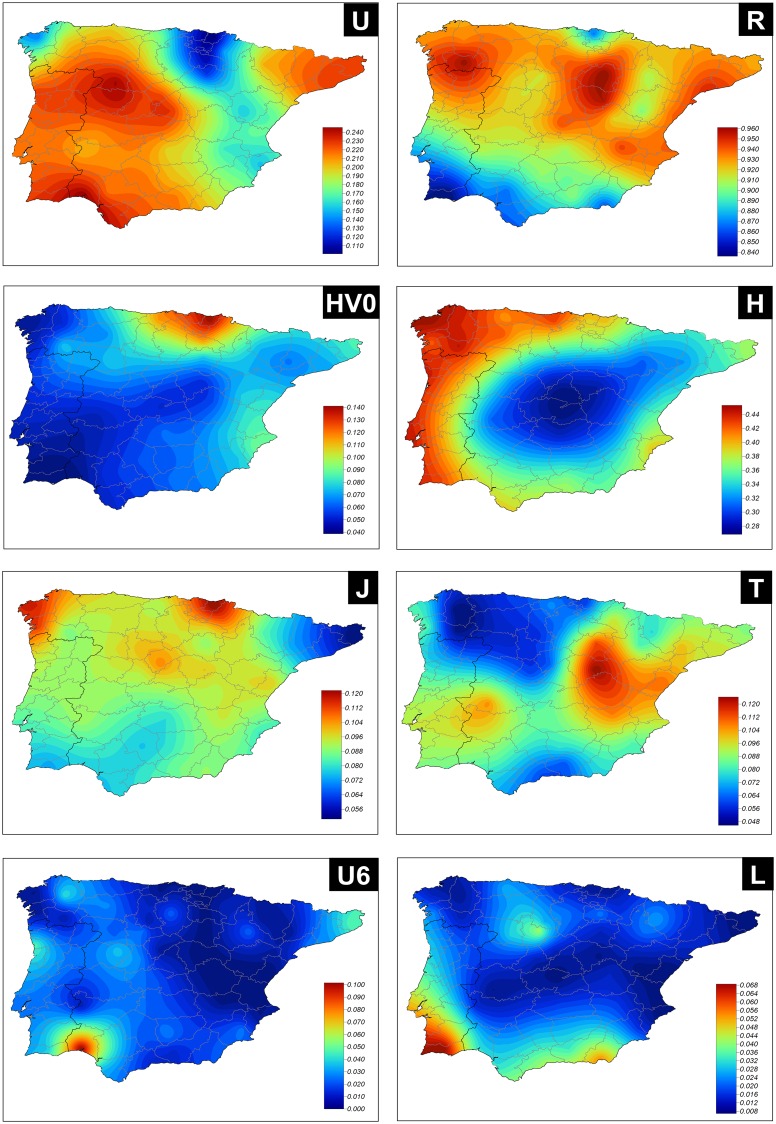
Geographic maps of the haplogroup frequencies. See legend of Fig 1 for more information.

Haplogroup frequencies can also be examined by provinces. Interpretations of spatial patterns of variation can be facilitated by interpolating these frequencies to the whole Iberian territory (Fig 3). Haplogroup R is more frequent in the northern half of the Iberian Peninsula; whereas the lowest values are found in the South of Portugal and Spain (South of Andalusia). Haplogroup HV0 (which is largely represented by haplogroup V) is much more frequent in the Basque Country than in any other Iberian region. Haplogroup H, which is also nested within haplogroup R, has however a different overall distribution; it spreads all along the Atlantic coast (including the Cantabrian Sea) with decreasing values towards the Mediterranean coast. Haplogroup J is more frequent in the northwest corner of Spain and in the Basque country, while its sister haplogroup T is more frequent in the Mediterranean coast. Finally, the interpolated map of the sub-Saharan haplogroup L shows its highest frequency in the South, as it also occurs with the North African haplogroup U6.

### AMOVA

Analysis of population subdivision using AMOVA reveals that most of the variation occurs within populations when computed either by main geographic regions (99.78%) or by political regions (99.69%); Table 4. Only a small (but statistically significant, *P*-value < 0.0000) proportion of the total variation can be attributed to variation among groups, and among populations within groups, when computed by either the main geographic regions or by Autonomous Regions (Table 4).

**Table 4:** Analysis of molecular variance (AMOVA) accounting for main geographic regions and Autonomous Regions (*P*-value < 0.0000).

Source of Variation	Percentage of Variation	
	Main geographic regions	Autonomous Regions
Among groups	0	0.15
Among populations within groups	0.30	0.16
Within populations	99.78	99.69

### New branches of the mtDNA phylogeny

**Fig 4:**
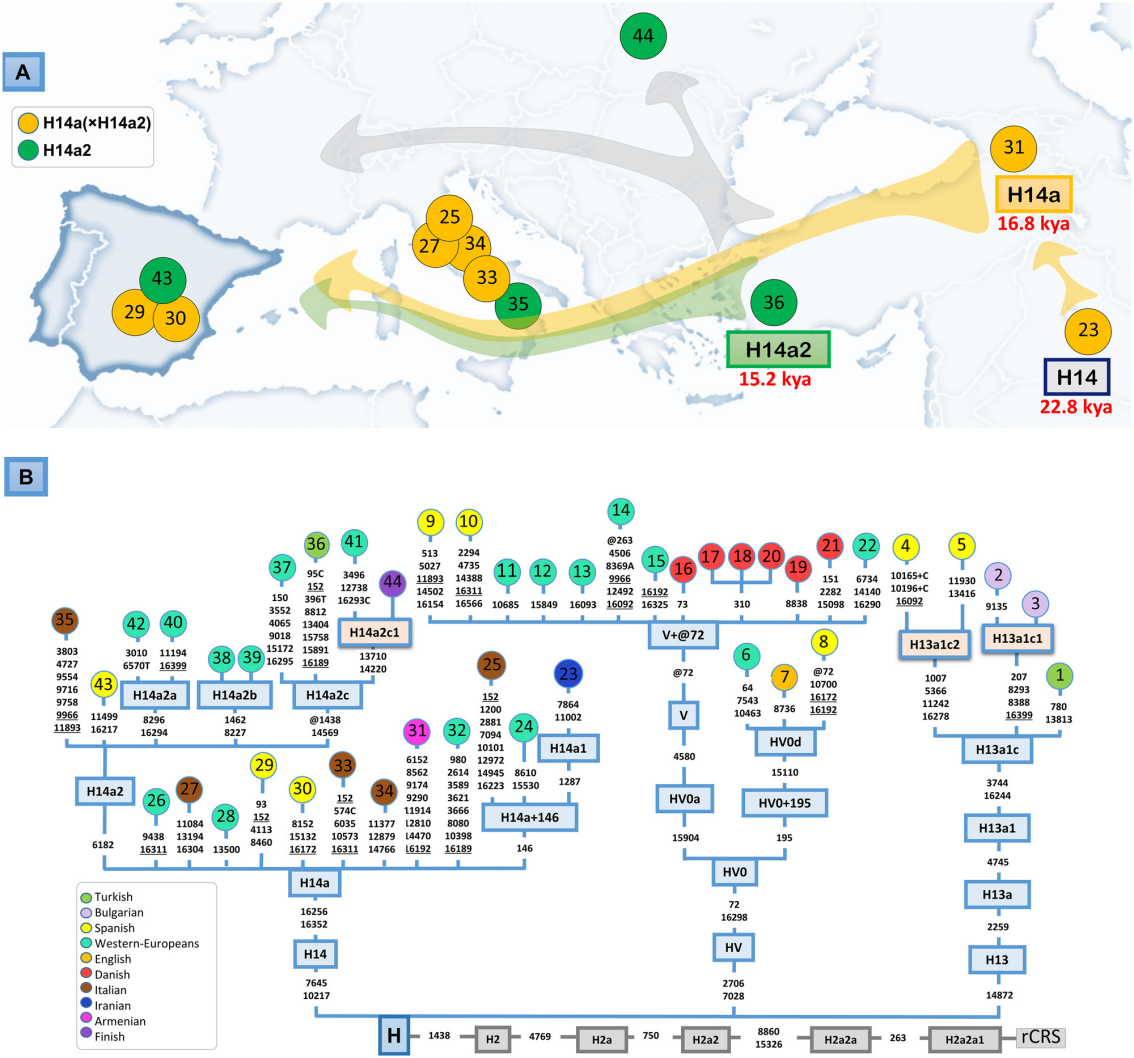
(A) Dispersal of H14a and H14a2 haplogroups along the Mediterranean coast. (B) Maximum parsimony tree of haplogroups represented by the Iberian mitogenomes sequenced in the present study. The position of the revised Cambridge reference sequence (rCRS) is indicated for reading sequence motifs [53]. Mitochondrial DNA variants are indicated along the branches of the phylogenetic tree. Mutations are transitions unless a suffix A, C, G, or T indicates a transversion. A suffix ‘+’ indicates insertions. Variants underlined represent recurrent mutations in this tree while a prefix ‘@’ indicates a back mutation. Mutational hotspot variants at positions 16182, 16183, and 16519, as well as variation around position 310 and length or point heteroplasmies were not considered for the phylogenetic reconstruction. The color of the circles indicates their geographic origin according to the legend inset. The Iberian complete genomes obtained in the present study are indicated with yellow circles. More details on the geographic or ethnic origin of all the mitogenomes used in this network are provided in S3 Table. Haplogroups in orange boxes represent new branches of the mtDNA phylogeny, and are therefore not present in Phylotree Build 17.

A few control region haplotypes were selected from the whole dataset for complete genome sequencing. The criterion for selection was the existence of a characteristic control region motif, that is, a motif that does not fit with known branches of the phylogeny. With this strategy we were able to identify a few new minor clades (Fig 4). Haplogroup H13a1c1 is defined by two Bulgarian samples that carry the diagnostic motif G207A–C8293T–T8388C–A16399G on top of the H13a1c motif. Its sister clade H13a1c2 is defined by two Spanish samples carrying the motif G1007A–C5366T–C11242T–A11278G.

Samples #9 and #10 fit within the V sub-clade that lost the known diagnostic substitution T72C located in HVS-II (V+@72); Fig 4. This finding highlights the fact that T72C varies within haplogroup V.

Spanish sample #8 falls within HV0d (defined by transition G15110A) together with two other European haplotypes (Fig 4).

Samples #29, #30 and #43 carry the H14a known HVS-I motif C16256T–T16352C (Fig 4). There are 22 mitogenomes within haplogroup H14a; H14a2 is the main sub-clade and it incorporates nine members sharing transition G6182A; one of them is Spanish sample #43. Haplogroup H14a is mostly found in Western Europe. Its phylogeographic patterns suggest an ancient demographic connection between Near East and Western Europe, following a dispersal of its members all along the Mediterranean coast (including one Iranian, one Turkish, one Armenian, one Bulgarian, five Italian, and three Spaniards). TMRCA for H14 is 22.8 thousand years old (kya), and for H14a it is 16.8 kya; while for its main sub-clade, H14a2, it is 15.2 kya (Table 5).

**Table 5:** TMRCA for haplogroup H14 and its sub-lineages.

Haplogroup	Genetic distance	S.E.	Mean (ky)	95% CI
H14	8.31	1.76	22.8	13.0–33.1
H14a	6.23	0.85	16.8	12.2–21.6
H14a2	5.67	0.91	15.2	10.3–20.3
H14a2a	2.74	1.23	7.2	0.9–13.8
H14a2c	5.67	1.26	15.2	8.4–22.3
H14a2c1	1.67	1.10	4.3	0–10.1
H14b	5.13	0.98	13.7	8.5–19.1
H14b1	0.44	0.44	1.1	0–3.4
H14b2	3.65	1.13	9.7	3.7–15.8
H14b3	3.21	1.16	8.5	2.4–14.7
H14b4	3.49	1.35	9.2	2.2–16.6

## Discussion

An important effort of compiling mtDNA sequencing data from Spain and Portugal was carried out for the present study. Data from the literature were supplemented with a significant amount of newly generated control region sequences. Iberia is one of the most thoroughly sampled regions (in Europe and probably worldwide) analyzed for mtDNA variation, considering its relatively small size.

Patterns of molecular diversity by main Iberian geographic regions, Autonomous Spanish regions, or provinces are difficult to interpret when examining one by one; however, interesting patterns of spatial stratification emerge when this variation is examined globally. It is important to note that the resolution of the control region is limited for the classification of some sequences belonging to certain haplogroups (e.g. H); however this strategy allows us to reveal patterns of haplogroup geographic variation that would remain unnoticed using other methods (e.g. by computing molecular diversity indices). Therefore, although some haplogroup categories could be better defined on the basis of coding region data, the use of the same sequence range for all the analyses and a homogeneous haplogroup classification methodology (Haplogrep) allow the gathering of an unprecedented large sample size, and fine-grained geographic representation of the Iberian Peninsula. This effort allowed comparisons of molecular variation patterns that would otherwise not be possible.

Examining patterns in main haplogroup categories revealed the existence of important geographic substructure in the Iberian Peninsula. For instance, haplogroup R is more prevalent in the northern half of the Iberian Peninsula than in the South. In good agreement with previous literature, haplogroup HV0 (which contains haplogroup H and V) finds a peak frequency in the Basque country region, adding support to the theory of this region being part of the Franco Cantabrian refuge, from where these lineages experienced re-expansion towards the rest of Europe after the LGM. The substantial geographic isolation of this region from the rest of Iberia would explain the preservation of these genetic features in present-day populations.

Haplogroup H, a cluster that is nested within haplogroup R category, is more prevalent along the Atlantic facade, including the Cantabrian coast; it displays the highest frequency in Galicia (northwestern corner of Iberia). The frequency of haplogroup H shows a decreasing trend from the Atlantic facade towards the Mediterranean and Andalusian regions. This finding adds strong evidence to the pioneering finding by Salas et al. [16], where Galicia was found to be a *cul*-*de*-*sac* population, a kind of European edge for a major ancient central European migration. Therefore, there is an interesting pattern of genetic continuity existing in the Cantabrian coast (also extending to Portugal), a pattern that has been observed previously when minor sub-clades of the mtDNA phylogeny were examined [12].

Haplogroups J and T show also some distinctive geographic patterns. While J is more prevalent towards the northwestern corner of the Iberian Peninsula and the Basque Country, haplogroup T shows the opposite pattern, that is, a higher prevalence in the Mediterranean area.

While the patterns described for haplogroups R, HVO, H, J, and T were probably set in Mesolithic and Neolithic times [43], patterns of haplogroup U6 and L are mainly explained by more recent demographic events. For instance, the Arab conquest of the Iberian Peninsula that resulted in the destruction of the Visigothic Kingdom and the establishment of the independent Emirate of Córdoba under Abd-ar-Rahman (years 756–929), could have contributed to raise the frequency of this haplogroup in this region. The Arab influence was however restricted to the southwest of Andalusia, in the bordering region with southern Portugal.

L-haplotypes are more frequent in the South of the Iberian Peninsula. The documented role of Portugal and Spain in the transatlantic slave trade (a process that involved about 12 million African slaves) [44,45] could have contributed to raise the frequency of this clade in Iberia. This pattern however could also be related to the Arab conquest of the Iberian Peninsula or to more ancestral demographic movements [46].

The present study reveals the existence of geographic patterns of mtDNA variation in the Iberian Peninsula. The data provide further support to the debated role of the Franco Cantabrian region as a refuge of European variation during the LGM period.

Last but not least, the existence of population substructure in the Iberian Peninsula might be relevant in biomedical studies. Medical studies have shown an interest in the implications of mtDNA haplogroup in complex diseases. There is a large body of literature aimed to assess the increased genetic susceptibility of some haplogroups to a number of complex diseases. While some of these findings could represent real biological associations, a few studies have proposed that these findings might just represent false positives, with population substructure being a risk factor of positive association in case-control studies [3,7–9]. The forensic genetics community is also aware of the implications that population substructure could have on the weight of the mtDNA evidence given that this is highly dependent on haplotype and haplogroup frequencies [1,47–49]; see also the recommendations of the DNA commission of the International Society of Forensic Genetics [1].

The present study has also made an effort in characterizing new branches of the mtDNA phylogeny, by way of sequencing the complete genome of some selected lineages that have a sequence motif in the control region. The newly defined branches are nested within haplogroup R, and would also set the basis for future investigations, such as those that revealed the existence of particular patterns of variation in the Franco Cantabrian refuge. Haplogroup H14a is particularly interesting. The TMRCA of this clade is approximately 16.8 kya. Its phylogeographic distribution indicates the presence of H14a members mainly in the Mediterranean European coast. Overall, its phylogeographic distribution and TMRCA suggest that H14a might have originated in the Near East, and that it travelled westwards towards Iberia following a mainly Mediterranean route well before the Neolithic expansions into Europe, in a pre-LGM period (Fig 4). This would agree with previous evidence indicating a Middle Eastern origin for H14 more than 22 kya [50,51].

Finally, spatial variation was analyzed using summary molecular diversity indices and AMOVA. However, these tools did not capture patterns of variation as clearly as they were revealed by phylogeographic investigations. It can be tentatively proposed that the limited sensitivity of these measures to infer or measure population subdivision might render them unsuitable for some biomedical applications. In forensic genetics, for instance, *F*_*ST*_ are generally recommended for correcting the weight of the DNA evidence; some critical assessments however suggested that *F*_*ST*_ could be inadequate when applied to individual profiles in real casework [6,52]. The present study indicates that local sampling might be more convenient for forensic evaluations than global databases. The main implication of the present study for medical investigations is that special care should be taken in geographically and ethnically matching cohorts of cases and controls in association studies carried out in the Iberian Peninsula.

## Supporting Information

S1 Fig Haplogroup frequencies by (politically-defined) Spanish Autonomous Regions. See legend of Fig 1 for more information.
(TIF)

S1 Table Mitochondrial DNA sequences obtained in the present study. (XLSX)

S2 Table Mitochondrial DNA sequences compiled from the literature and used for population comparisons. (XLSX)

S3 Table Complete mtDNA genomes obtained in the present study and those (collected from the literature and databases) used to generate the phylogenetic tree in Fig 4. (XLSX)
