# Supplementary figures and images for: Meta-Analysis of Mitochondrial DNA Variation in the Iberian Peninsula
Source: PLoS One. 2016 Jul 21;11(7):e0159735. doi: 10.1371/journal.pone.0159735 (PMC4956223; doi:10.1371/journal.pone.0159735)

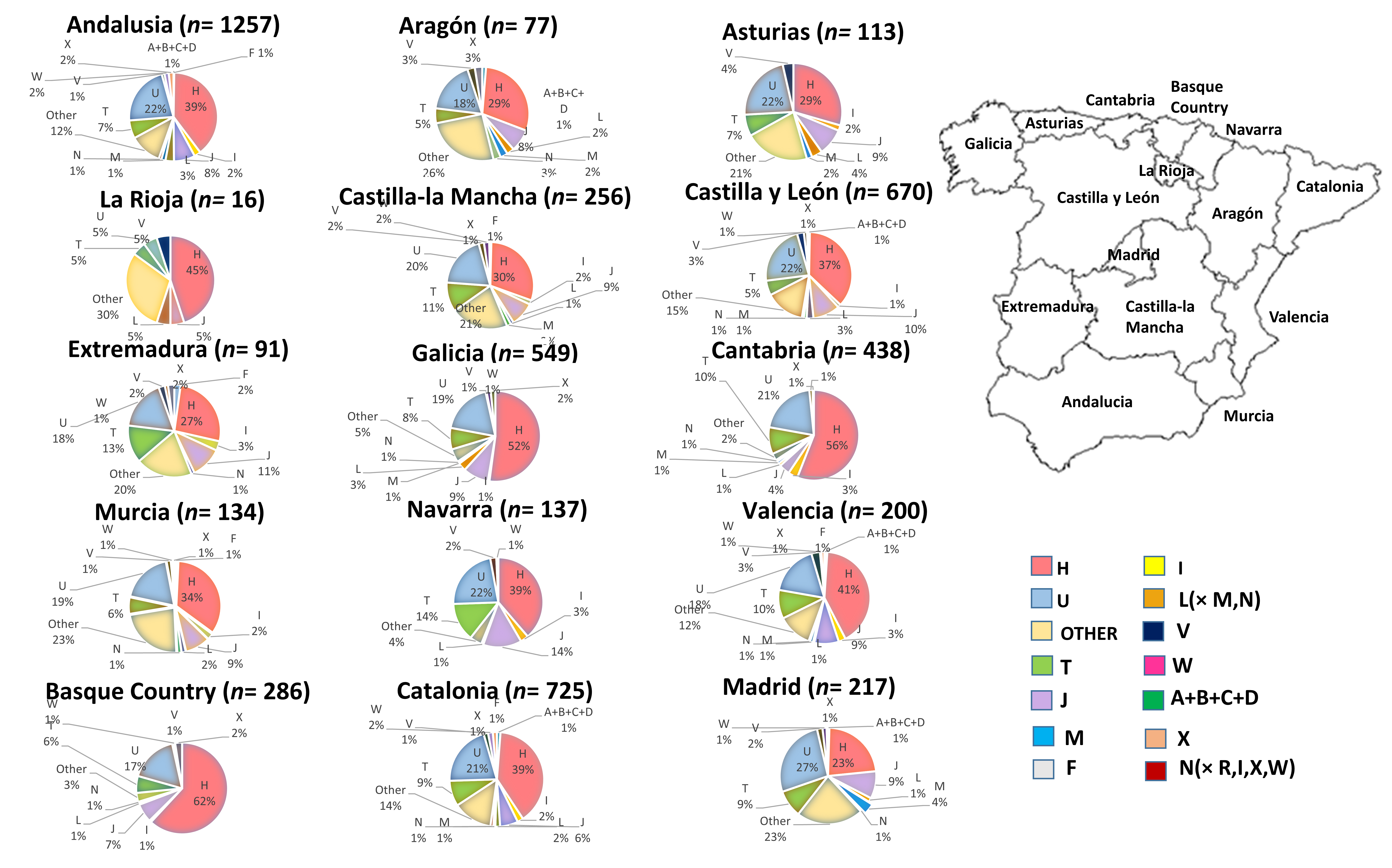

Supplement: S1 Fig — See legend of Fig 1 for more information. (TIF) [file pone.0159735.s001.tif]
